# Emerging Opportunities for Serotypes of Botulinum Neurotoxins 

**DOI:** 10.3390/toxins4111196

**Published:** 2012-11-07

**Authors:** Zhongxing Peng Chen, J. Glenn Morris, Ramon L. Rodriguez, Aparna Wagle Shukla, John Tapia-Núñez, Michael S. Okun

**Affiliations:** 1 University of Florida Center for Movement Disorders & Neurorestoration, Department of Neurology, 3450 Hull Road Gainesville, FL 32607, USA; Email: zhong.pengc@gmail.com (Z.P.C.), ramon.rodriguez@neurology.ufl.edu (R.L.R.); aparna.shukla@neurology.ufl.edu (A.W.S.); 2 ATIX Foundation, Avda. 11 de Septiembre 1363, office 1502, Santiago, Chile; Email: jtapian@hotmail.com; 3 University for Development, Av. Plaza 680, San Carlos de Apoquindo, Las Condes, Santiago, Chile; 4 University of Florida Emerging Pathogens Institute, 2055 Mowry Road Gainesville, FL 32610, USA; Email: jgmorris@epi.ufl.edu

**Keywords:** Botulinum toxin serotypes, neurotoxins subtypes, neuro-pharmacology toxins

## Abstract

Background: Two decades ago, botulinum neurotoxin (BoNT) type A was introduced to the commercial market. Subsequently, the toxin was approved by the FDA to address several neurological syndromes, involving muscle, nerve, and gland hyperactivity. These syndromes have typically been associated with abnormalities in cholinergic transmission. Despite the multiplicity of botulinal serotypes (designated as types A through G), therapeutic preparations are currently only available for BoNT types A and B. However, other BoNT serotypes are under study for possible clinical use and new clinical indications; Objective: To review the current research on botulinum neurotoxin serotypes A-G, and to analyze potential applications within basic science and clinical settings; Conclusions: The increasing understanding of botulinal neurotoxin pathophysiology, including the neurotoxin’s effects on specific neuronal populations, will help us in tailoring treatments for specific diagnoses, symptoms and patients. Scientists and clinicians should be aware of the full range of available data involving neurotoxin subtypes A-G.

## 1. Introduction

Over the past three decades, there has been a continuing fascination with botulinum neurotoxin (BoNT) and the opportunities for use of this potentially deadly neurotoxin as a treatment in certain clinical settings. BoNT interferes with the liberation of acetylcholine from presynaptic nerve endings, and clinically the therapy results in the prevention of muscle contractions and/or gland secretion. Since BoNT is used primarily as a focal injectable treatment, resulting in fewer side effects than systemically administered medications, it has evolved to become the preferred treatment of choice for management of many forms of dystonia, limb spasticity, cosmetic glabellar lines, hyperhidrosis, and sialorrhea [1]. The use of OnabotulinumtoxinA to address migraines was approved by the FDA in 2010 [2], and its use to treat bladder hyperactivity was approved in 2011 [3,4,5]. BoNT also has a broad spectrum of off-label indications, including focal hand dystonia, lower limb spasticity [6,7], management of chronic anal fissures [4], and tension headaches [8]. The commercially available neurotoxins include three main brands of BoNT/A: OnabotulinumtoxinA (BOTOX^®^), AbobotulinumtoxinA (DYSPORT^®^), IncobotulinumtoxinA (XEOMIN^®^), and also a brand of BoNT type B, RimabotulinumtoxinB (MYOBLOC^®^ or NEUROBLOC^®^) [9]. Each formulation has specific clinical recommendations which have been formulated based on the available science, and on differing biochemical profiles. Much of the literature has focused on the A and B neurotoxin types, with little mention of the other seven serologic types. Our goal in this review paper was to update the latest biomolecular findings in BoNT science. This review will cover the clinical scope, including mechanisms of action, and also will summarize the neurotoxin properties of the seven different toxin serotypes (A-G). Finally, we will discuss the potential opportunities for utilizing the various serotypes in clinical settings.

## 2. *Clostridium botulinum* and Botulism: Botulinum Neurotoxin as a Cause of Disease, and as a Medication

Botulinum neurotoxins (BoNT) are protein complexes produced by anaerobic gram positive Bacilligrouped under the name of *Clostridium botulinum*. The first description of botulism was penned in 1822 by Justin Kerner, who described a group of people suffering muscular weakness following the ingestion of contaminated sausages [10].

*C. botulinum* strains/species produce spores that are ubiquitous in the environment, surviving for extended periods of time under adverse environmental conditions. Under appropriate anaerobic conditions, spores may germinate, and the resultant vegetative cells may produce neurotoxin. Spore germination and neurotoxin production has been described in foods and in association with improper canning (particularly home canning); inappropriate fermentation of meat/sausage, bean curd, and fish; and preservation of a variety of products under oil or conditions that promote an anaerobic environment [11]. Much of the current food regulatory structure in the United States is based on assuring that conditions under which food is prepared and stored do not facilitate germination of *C. botulinum* spores and neurotoxin production.

The ingestion of food contaminated with pre-formed BoNT, or, more rarely, infection by *C. botulinum* of an exposed wound or growth in an immunologically immature intestinal tract (*i.e.*, infant botulism) [12], can produce the clinical syndrome of botulism. Human botulism has been associated with BoNT types A, B, E and F, with BoNT/F being the least frequently reported [13]. Types C and D have been associated with botulism in animals. In accordance with the observed geographic distribution of botulinum spores in the environment, type A cases in the United States have come predominantly from areas west of the Mississippi, while type B cases predominate in the eastern United States. Type E cases are generally associated with fish or seafood, and, in the United States, tend to be associated with the consumption of fermented fish from Alaska [14].

Botulism is characterized by progressive flaccid paralysis of motor and autonomic nerves, and it usually occurs in a proximal to distal pattern, starting from muscles innervated by the cranial nerves, and then moving sequentially to affect the upper limbs, followed by respiratory muscles, and ultimately the lower limbs. Severe cases of botulism can include respiratory muscle paralysis, leading to ventilatory failure and death, if immediate supportive care is not provided [15]. Once clinical symptoms appear, the neurotoxin is considered to have irreversibly bound to receptors and entered the cell, blocking acetylcholine release. The administration of antitoxin can neutralize unbound neurotoxin, but cannot reverse existing symptoms [13]. 

Emile Piere Van Ermergen isolated the crystalline form of the neurotoxin type A in 1895, and the clinical product of BoNT/A was not produced until 1946 by Edward Schantz [10]. The neurotoxin was first used in 1981, by Alan Scott (an ophthalmologist) for a patient in his practice with strabismus [10]. In subsequent years, these uses have multiplied, and now include dystonia, spasticity, sweating, cosmetics, and many other indications. 

## 3. Botulinum Neurotoxin Structure Serotypes and Subtypes

### 3.1. Structure

**Figure 1:**
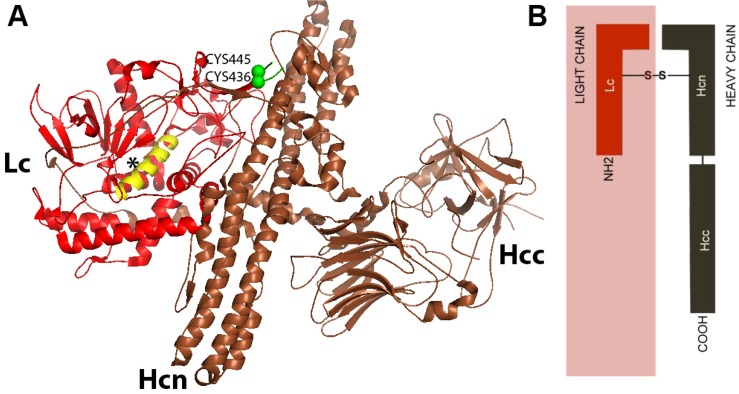
(**A**) Illustration of molecular structure of Botulinum Neurotoxin type B (BoNT/B). Botulinum neurotoxin is composed of a heavy chain (Hc) attached to a light chain (Lc) by a non-covalent disulfide bridge (Cys^445^–Cys^436^). Hc is divided in an amino region (Hcn) and a carboxyl region (Hcc). Hcc is the binding domain; Hcn is the translocation domain, and Lc is the catalytic domain [16].The asterisk (*) indicates the helix of Lc containing the motif HEXXH;(**B**)A simplified illustration of the BoNT/B structure. Figure 1A was modified from the RCSBProtein Data Bank [20].The molecular modeling was refined with *PyMOL Molecular Graphics System*, *Version 1.2r3pre*, *Schrödinger*, *LLC.*

**Figure 2:**
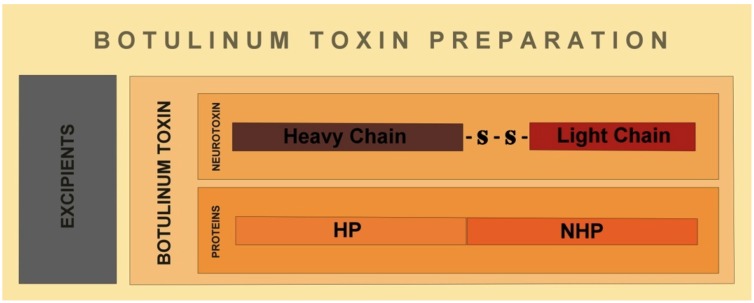
Commercial preparations of botulinum neurotoxins include the botulinum neurotoxin molecule, and non-toxic haemagglutinin proteins (HP) and non-haemagglutinin proteins (NHP), plus excipients.The associated proteins are thought to stabilize the neurotoxin [21].*Proteins complexes are not present in IncobotulinumtoxinA, which is a formulation of botulinum toxin serotype A [22].

The botulinum neurotoxin is produced as a 150 kDa polypeptide, and contains a heavy chain of 100 kDa (Hc) which is attached to a light chain molecule of 50 kDa (Lc). The attachment is by a disulphide non-covalent bond [16]. The heavy chain (Hc) is divided into an amino domain (Hcn) and a carboxyl terminal domain (Hcc) (Figure 1) [17]. While the Hcn portion is highly homologous among various clostridium neurotoxins, the Hcc presents more variability [18]. At the same time, the light chain (Lc) of all BoNTs has an extremely conserved region called HEXXH, which consists of zinc binding motifs of zinc endoproteinases [19]. Lc acts as a zinc dependent metalloprotease that is directed to cleave specific proteins from a soluble *N*-ethylmaleimide-sensitive factor activating protein receptor (SNARE) complex, in order to prevent the release of acetylcholine into the synaptic cleft [16]. The neurotoxin, composed by the Hc and the Lc, is associated with non-toxin proteins, which possess haemagglutinin properties [HP] and non-toxic proteins lacking haemagglutinin properties (NHP) (Figure 2). 

The encoding genes of BoNT and its proteins are localized in different positions in the *C. botulinum* genome, and vary depending on the individual serotypes. The transcription of the neurotoxin is regulated by the gene *botR*, which produces a protein of 21 kDa. The protein possesses 67% homology to the *tetR* product of Clostridium Tetani [23]. The toxin produced by *Clostridium Tetani* (TeTx) presents similar structures and mechanisms of action compared with botulinum neurotoxin, and it is synthesized also as a single-chain protein of ~150 kDa which is proteolytically activated to di-chain derivatives involving a Lc of ~50 kDa, also linked by a single disulfide bridge to a Hc of ~100 kDa. However, in contrast to botulinum neurotoxin, the TeTx does not form protein complexes [24]. Molecular studies have found highly conserved genes between *C. tetani* and *C. botulinum* which are functionally interchangeable, suggesting a possible common ancestor.

The complete structure of the BoNT complexes has not been fully elucidated. These complexes are classified according their sizes [17,24,25,26]. M complexes are 230–350 kDa and are composed of the 150 kDa BoNT associated with non-toxic non-haemagglutinin proteins (NHP) of about 120–140 kDa. BoNT types C, D, E and F are attached in M complexes, L complexes are 450–500 kDa, consisting of M complexes associated with non-toxic haemagglutinin proteins (HP). BoNT type A and B form L complexes. BoNT/A can be associated with dimers of L complex components, and can reach sizes of 900 kDa. The protein complexes dissociate at pH levels >7, releasing the free neurotoxin to act over its target. At pH values of 5–7, the protein complexes remain stable. The associated proteins have traditionally been thought to protect the neurotoxin at low environmental pH’s and also protect it from proteases [27]. However, there are recent studies in animals suggesting that protein complexes may not be essential for neurotoxin stability [28]. The proteins may also facilitate neurotoxin absorption into the lymph and circulatory system [13,25,26].

### 3.2. Serotypes

While having the format of a species name, the term *C. botulinum* is best regarded as a loose taxonomic descriptor for a group of strains/species that have in common the ability to produce botulinum neurotoxins. Based on physiologic characteristics and genetic backgrounds, these strains cluster into 4 groups (designated I-IV; Table 1). Genetic differences among groups are sufficient to justify designation of each group as a separate species. Each group tends to cluster with certain other, previously identified clostridial species, and strains within each group produce a limited repertory of neurotoxin types, the genes for which appear to have been acquired independently through horizontal gene transfer [29,30]. Seven serologically distinct neurotoxins (designated as neurotoxin types A-G) are produced by strains/species within these four groups. Sequence analysis of neurotoxin genes, together with amplified fragment length polymorphism (AFLP) analysis, results in a further subdivision of neurotoxin types, including four distinct lineages within each of the BoNT A and B serotypes, and five distinct lineages of serotype E strains [29]. 

Although all of the seven serotypes (A, B, C, D, E, F and G) can potentially produce botulism in a human, only BoNT type A, B, E and rarely BoNT type F, have been documented to affect humans [12,31]. *Clostridium botulinum* type A is responsible for the highest rate of mortality of botulism in humans. Its recent genomic analysis has revealed that its plasmid does not harbor neurotoxin gene(s), but rather genes participating in the replication and encoding of ABC type multidrug transport ATPase and permeases, suggesting the participation of efflux pumps in bacteriocin production, modification and export in *C. botulinum*. Genes responsible for genomic rearrangement seem also to be involved [32].

**Table 1:** Botulinumneurotoxin: Strain groups and neurotoxin serotypes. There are four phenotypic groups (I to IV) [33]. Group I includes proteolytic strains (with ability to breakdown proteins), that produce one or more types of BoNT (A, B or F). Group II are non-proteolytic strains (without the ability to breakdown proteins) that produce only one type of BoNT (B, E or F). Group III strains produce BoNT type C or D, and finally group IV strains produce BoNT type G. Phylogenetic analyses based on BoNT protein sequences have revealed a close relationship between neurotoxins A and E, between neurotoxins C and D, and among neurotoxins F, G and B [32,34].

Group	Toxin types produced	Additional strains closely related *Clostridial* species
I	A, Proteolytic B, F	*C. Sporogenes*
II	E, Non-proteolytic B, F	*C. Novyi*, *C. haemolyticum*
III	C, D	*C. Subterminale*, *C. Proteolyticus*
IV	G	*C. Argentinense*, *C. Schirmacherense*

### 3.3. Subtypes of Neurotoxin Serotypes

There are subtypes for each of the A-G varieties of botulinum neurotoxins. The criterion for a subtype is based on a ≥2.6% difference in amino acid sequences [35]. The variation in amino acid sequences in the BoNT can determine significant differences in the immunological and biological properties of the neurotoxin [36]. 

Five subtypes of BoNT/A have been described A1–A5, but only A1, A2 and A5 have been purified and analyzed at a protein level to determine functional differences [35]. All five subtypes of BoNT/A have a similar affinity for binding SNAP25, but BoNT/A3 and BoNT/A4 cleave SNAP25 with much less efficiency than BoNT/A1 and A2 [37]. BoNT/A1 and BoNT/A2 differ in approximately 10% of their amino acid sequences. The main difference has been identified at receptor binding domains. BoNT/A assays in cultured neurons have been observed to have more rapid translocation of BoNT/A2 into cells, compared with A1, which confers a greater biological potency than BoNT/A1. The duration of action has been observed to be similar in both subtypes of BoNT/A [38]. BoNT subtype A5 (BoNT/A5) strains have been observed to have differences, as compared to the BoNT subtype A1 [35].

BoNT/B has also been divided into subtypes B1, B2 and B3 [36]. The amino acid differences were found in nucleotide sequences which partially encoded the Hc carboxyl sub-domain (Hcc). Also, recent studies have shown six subtypes of BoNT/E named as E1, E2, E3, E4, E5 and E6 [39]. The nucleotide sequences of BoNT/E had less amino acid variability than BoNT/A, but showed enough differences to affect neurotoxin biological and antigenic properties. The last BoNT/E subtype was E6, which differed 3% to 6% from other subtypes in amino acid sequence. Specific features of neurotoxin types and subtypes have been appearing in the recent literature since mild variations of neurotoxin nucleotides can change structure, function and antigenic properties [40,41,42].

## 4. Measuring the Potency of the Botulinum Neurotoxins

The gold standard measurement of BoNT potency has been defined as the median lethal dose of the neurotoxin needed to cause death of 50% of a population of Swiss Webster Mice (LD50) [43,44,45]. The LD50 values for mice range from 0.5 to 5 ng/kg depending on the serotype [17]. Alternatively, potency may be reported as LD50’s per mg of toxin. However, minor differences in testing procedures, such as the strain of mice used, or the choice of dilution buffer, can result in different LD50 values for an essentially identical sample. Because of the differences, the units used in labeling the neurotoxin potency are specific for each marketed product, and are considered not to be interchangeable. 

Another way to test neurotoxin potency is the mouse abdominal ptosis assay [45,46]. This assay measures the dose of neurotoxin injected subcutaneously into the top of a mouse hind leg. The procedure is designed to produce weakness in the animal. The animals are scored using a five point scale, according to the abdominal bulge present at 24 and 48 h. The potency of neurotoxin can then be estimated using a parallel line method.

## 5. Mechanism of Action of Botulinum Neurotoxins

Physiologically, the neuromuscular transmission begins with an action potential arriving at the presynaptic endplate, which then transiently depolarizes the axon terminal, and briefly opens the calcium channel. The increase of intracellular calcium leads to exocytosis of Ach containing vesicles into the neuromuscular junction. Two molecules of Ach are required to bind Nm nAChR to open its central ion channel and to permit sodium to diffuse into the muscle cell, thereby depolarizing the endplate. The endplate potential spreads to nearby muscle fiber membranes and depolarizes them to threshold, and this initiates a muscle cell action potential. The propagation of this action potential along the terminal axon of the motoneuron induces the release of acetylcholine from the cytosol into synaptic clefts by the Soluble *N*-ethylmaleimide-sensitive factor activating protein receptor complex (SNARE). Once in the synaptic cleft, the acetylcholine molecules bind to post synaptic membrane and muscle fiber contraction is the resultant effect [47].

Biomolecular research has been providing important clues to assist in the understanding of the mechanism of BoNT action. In general terms, the carboxyl terminal of the heavy chain (Hcc) binds to specific receptors from the presynaptic terminal. BoNT is endocytosed and then the amino domain of the heavy chain (Hcn) translocates the Light chain (Lc) into the cytoplasm. Once in the cytoplasm, the light chain (Lc) is directed to cleave a determinate membrane or vesicle associated proteins, thus preventing acetylcholine release.

The process can be subdivided into four stages: (1) binding; (2) internalization; (3) membrane translocation; and (4) proteolysis of specific SNARE proteins.

### 5.1. Binding

**Figure 3:**
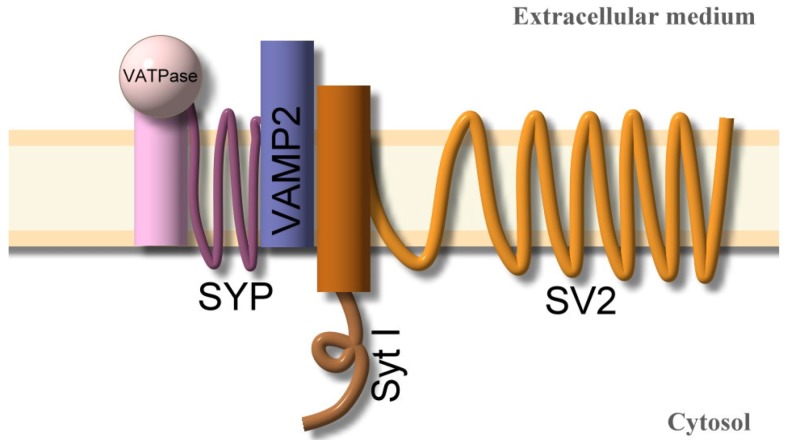
Array of proteins associated with the presynaptic vesicle membrane [32]. The figure illustrates a suggested complex array of proteins that are anchored to the membrane of the vesicle that is exposed to the presynaptic terminal during the process of vesicle recycling.

The nerve terminal membrane contains multiple molecules in an array of presynaptic receptors (Figure 3), which can bind the seven types of BoNTs via a combination of communal and serotype specific receptor molecules (Table 2). The neurotoxin has an active site of binding localized in the carbonyl terminal of the heavy chain (Hc). This region recognizes and binds to specific gangliosides and vesicle associated proteins (SV). These are present on the inner surface of the presynaptic vesicle during their exposure to an external medium. 

**Table 2:** Botulinum neurotoxin serotypes receptors [48,49,50]. **White area**, The heavy chain of neurotoxin binds to a presynaptic protein receptor array, comprising gangliosides (GD1a, GD1b, GT1b, GQ1a, GM1a) and specific proteins including synaptotagmins (Syt-I, Syt-II) and synaptic vesicle associate proteins (SV2A, SV2B, SV2C);**Gray area** shows the selective prevalence of Syt and SV2 protein isoforms at the neuromuscular junction, and in the hippocampus excitatory and inhibitory neurons. SV2A and SV2B are prevalent in the hippocampus excitatory neurons and SV2A and SV2C are both prevalent in hippocampus inhibitory neurons. All SV2 are equally present in the neuromuscular junction. Syt-I predominates in the hippocampus excitatory and inhibitory neurons, and Syt-II is more commonly detected at the neuromuscular junction and in excitatory neurons of the hippocampus. Binding affinity of BoNT/A is SV2C>SV2A>SVC2B. BoNT/B has higher affinity to Syt-II and BoNT/G has higher affinity to Syt-I. BoNT/D, E and F bind to SV2 isoforms. The specificity of gangliosides binding neurotoxin subtypes is being studied, and increasing isoforms from Syt and SV are being described.

	BoNT (Receptors)	BoNT serotype Binding and Affinity to Presynaptic proteins							Neuromuscular junction [51,52]	Hippocampus [52,53] cortex excitatory neurons	Hippocampus [53] cortex inhibitory neurons
		A [54,55]	B [56,57]	C [58]	D [58,59]	E [49,60]	F [61]	G [62,63]			
**Proteins**	Syt-I	+	+				-	+	-	+	+
	Syt-II		+				-	+	+	+	
	SV2A	+	-		+	+	+		+	+	+
	SV2B	+	-		+	+	+		+	+	-
	SV2C	+	-		+	-	+		+	-	+
**Gangliosides**	GD1a		+			+	+				
	GD1b		+	+			-				
	GT1b	+	+		+	+	+	+			
	GQ1a										
	GM1a		+				-				
	GM3						+				

White area: (+) Presence of affinity of the protein/ganglioside to presynaptic proteins; (-) Absence or not significant affinity of the protein/ganglioside to presynaptic proteins; Blank spaces represent no information available at the moment to confirm or discard the presence or absence of protein/ganglioside binding affinity; Gray area: (+) Presence in the structure; (-) Absence in the structure.

The gangliosides are complex sialylated glycosphingolipids involved in the development, function and maintenance of the nervous system. They are abundant in presynaptic membranes [64]. Gangliosides seem to play a role in the sensitivity to BoNTs overall effects. Laboratory testing has shown that pre-treating cultured cells with gangliosides increases the sensitivity to BoNT/A, and that depletion of the polygangliosides in neuroblastoma cells has the opposite effect [17,65,66,67]. It has been suggested that the initial interaction between neurotoxin Hc and the presynaptic membrane is actually an interaction with the oligosaccharide domain of the ganglioside. The first step in the process seems to be reversible [68]. Upon binding of the neurotoxin to the membrane linked oligosaccharide, the neurotoxin loses its three dimensional structure, and changes to a bi-dimensional one, thereby decreasing the reaction volume. This binding enables the rotational and bending mobility of the moiety, which can facilitate additional membrane interactions [69].

BoNTs Hc fragments bind predominantly to subtypes of gangliosides and these bind according to neurotoxin serotype (Table 2). The presence of a conserved peptide sequence from BoNT/A, B and F, known as SXWY, seems to participate in gangliosides binding [62]. BoNT/E possesses a similar motif in which a lysine is replaced by a histidine [61,62]; However BoNT/D and C do not have this motif [70].

Recent studies suggest that the participation of external residues is necessary for stabilization of neurotoxin-ganglioside interactions, despite the conservation of the motif [70,71]. BoNT/B binding is facilitated by gangliosides GT1b > GD1a > GD1b > GM1 [72]. BoNT/A and BoNT/B binds to GT1b through the conserved motif SXWY. At the same time, BoNT/D can recognize gangliosides using either a ganglioside binding site (GBS) or protein binding site (PBS), and BoNT/C can recognize a ganglioside by what is thought to be a ganglioside binding site. GBS has an auxiliary contribution of a ganglioside binding loop (GBL) located between GBS and PBS [70].

Besides the polyganglioside recognition, some specific proteins associated with presynaptic membranes and vesicles are necessary for the Hc binding, and for endocytosis. The array of presynaptic receptors includes specific proteins: Synaptotagmin I (Syt-I) and II (Syt-II) and synaptic vesicle associated protein SV2A, SV2B, SV2C and VAMP2. Each protein has different affinities for individual BoNT serotypes (Table 2). BoNT/B interacts with the luminal domain of SV proteins; Syt-I and Syt-II and in the presence of GT1b to bind the presynaptic terminal; meanwhile BoNT/G interacts with both Syt, and does so independent of a ganglioside presence [62]. BoNT/A was reported to bind to the luminal domain of SV2, which is a highly glycosylated *trans*-membrane protein with no clearly defined function [54,55]. Moreover, neurotoxin receptor complexes contain several additional proteins including synaptophysin, synaptogyrin [73], and vacuolar ATPase (vATPase). Synaptophysin and synaptogyrin have been reported as redundant regulators of calcium dependent exocytosis [74], and vATPase is a multi-subunit trans-membrane protein which pumps protons from the cytoplasm to the lumen, thus acidifying the endosome [75].

### 5.2. Internalization

After the binding of the neurotoxin Hc with the ganglioside, lateral presynaptic membrane movements trap the neurotoxin inside the array of protein receptors, increasing its interaction with additional binding molecules, making it irreversible [69]. The internalization of neurotoxin involves trafficking via vesicles, by an energy dependent and temperature sensitive mechanism that is favored by the high rates of vesicle recycling from the hyperactive nerve terminal [48]. In physiological conditions, most of the synaptic vesicles are bound to the actin cytoskeleton of synaptic terminals via interactions regulated by protein phosphorylation. A small proportion of vesicles bind to the cytosolic face of the presynaptic membrane active zones, via protein–protein interactions [17]. The acetylcholine release is mediated by calcium triggered exocytosis of synaptic vesicles at the presynaptic active zone of nerve terminals. To support efficient and repeated rounds of release, synaptic vesicles undergo a trafficking cycle, which is hyperactive in nerves with increased acetylcholinergic activity. The recycling process leads to the docking and priming of the vesicles for another round of exocytosis and endocytosis [17].

### 5.3. Membrane Translocation

This is the stage of neurotoxin action that is least well understood. The most generally accepted model suggests that after neurotoxin endocytosis, the low pH of endosomes contributes to a conformational change of the light chain of the neurotoxin, exposing its hydrophobic segments to the surface [28,34]. This change enables the penetration of heavy and light chains in the hydrocarbon core of the lipid bi-layer of the vesicle [34,76], forming a membrane channel [17]. The amino terminal domains of the heavy chain participate in the translocation of the light chain into the cytosol, through the channel. It is not known how this is accomplished, as the architecture of the channel is unknown [77].

It has been suggested that an early low conductance state represents a partially occluded channel by the translocating Lc. The large conductance state at plateau may represent an unoccluded channel after Lc departs the channel and heads for the cytoplasm [78]. A recent study observed that ganglioside GT1b enabled BoNT/B to sense low pH, contributing to its oligomerization and translocation [77]. 

### 5.4. Proteolysis of Specific SNARE Proteins

After the release of neurotoxin light chain into the cytosol, it cleaves a distinct site on one or more proteins from the SNARE complex, preventing the release of acetylcholine into the synaptic cleft. These proteins are required for vesicle release. All the BoNTs have been described to have a Lc region containing an extremely conserved sub-region (HEXXH) which provides the zinc binding motifs for zinc endoproteinases directed to the SNARE complex [19,79]. Endoproteases cleave specific targets of the soluble *N*-Etylmaleimide attachment protein receptor (SNARE) (Table 3). Mechanisms are dependent on the serotype of the neurotoxin. The SNARE protein complex can be divided into the target membrane SNARE (SNAREt) which includes syntaxin and SNAP25 proteins, and the vesicle associated SNARE (SNAREv), also referred to as vesicle associated protein(s) (VAMP). BoNT/A, C and E cleave specific parts of SNAP25, located on the cytosolic face of the presynaptic membrane; BoNT/C can cleave specific parts of SNAP-25 and also syntaxin. BoNT B, D, F and G cleave part of SNAREv. The SNAREv includes isoforms of a protein associated with a vesicle membrane synt-I and synt-II and specific proteins from the luminal face of the presynaptic vesicle referred to as SV2A, SV2B, and SV2C. These proteins are found in varying prevalence in different tissues. All of the proteins contribute to neurotoxin specificity. Moreover, even if the same protein is affected, the different BoNT serotypes may have action at a different site, e.g., BoNT/A cleaves the amide bond between Gln^197^ and Arg^198^, and BoNT/E cleaves the bond between Arg^180^ and Ile^181^ [37]. Although the molecular bases of the catalytic activity of neurotoxins are not clear, it has been suggested that the ability for recognition and interaction of the neurotoxins Lc and for their SNARE protein substrates could determine their specificity [80]. The Lc of BoNT/A was described as recognizing SNAP-25 through non-covalent interactions between residues of neurotoxin and a region of SNAP-25 distant from the active site, aligning the P5 residue, Asp^193^, with Arg^177^, a S5 pocket of Lc to form a salt bridge [79]. In contrast, the Lc of BoNT/E substrate binding region comprises Leu^166^, Arg^167^, Asp^127^, Ala^128^, Ser^129^ and Ala^130^, and involves the participation of multiple pockets named as S1', S2 and S3 [80]. A recent study described the recognition of Lc of BoNT/B and TeTx of VAMP2 denominated as P7, P6, P1, P1', and P2, and fine alignment sites; P2 and P3 for BoNT/B and P2 and P4 for TeTx. Researchers found out that modification of the S1 pocket of TeTx Lc increased the rate of native VAMP2 cleavage, and it approached it the BoNT/B Lc rate, which highlight another potential target of work for future neurotoxin engineering.

**Table 3:** Botulinum neurotoxin serotypes profiles and cleavage target [81,82,83,84]. Potency of BoNT varies depending on serotype, with potency reported as LD50’s/mg toxin. The laboratory assay for neurotoxin duration is based on its proteolytic action (Hours-Days-Months). BoNT/A is the most potent serotype and has the longer acting proteolytic activity. Botulinum neurotoxin duration was estimated according its proteolytic activity in laboratory models. *In vivo* activity of BoNTs lasts 2–4 months from the injection. Information on BoNTs potency was obtained from *Metabiologics*, Inc. [85]. (+) Presence of action over target; (-) No action over target.

BoNT Type	*C. botulinum*	Laboratory measurement		Affects	SNARE Cleavage target			Commercial
					tSNARE		vSNARE	
		Potency LD50/mg	Duration *		SNAP 25	Syntaxin	VAMP	
A	Group I	3.7 × 10^7^	31 days	Human	+	-	-	Yes
B	Group I-II	1.2 × 10^7^	10 days	Human	-	-	+	Yes
C	Group III	5.8 × 10^6^	25 days	Animal	+	+	-	No
D	Group III	3.1 × 10^7^		Animal	-	-	+	No
E	Group II	3 × 10^7^	0.8 days	Human, fish	+	-	-	No
F	Group I-II	3.6 × 10^6^	2 days	Human	-	-	+	No
G	Group IV	4 × 10^6^		Human	-	-	+	No

Besides the Lc recognition of protein substrate and alignment, there has been a described interaction of internal pocket residues of BoNT/B and TeTx (S5, S4, S3, and S2) which participates in the physical orientation of the molecular structure to ensure an effective cleavage of the protein substrate [86]. Despite having a similar chemical structure, the different folding of the molecule can determine particular properties of the serotype, e.g., the faster toxic effect of BoNT/E compared with BoNT/A has been attributed to BoNT/E quick internalization and translocation, possibly related to its molecular structure conformation, in which the binding domain (Hcc) and the catalytic domain (Lc) are on the same side as the translocation domain (Hcn) [87]. This work, however, will need further study before it can be confirmed. Further work is needed not only on the specificity of the neurotoxin presynaptic recognition and internalization, but also on the specificity of the catalytic properties, the interactions between protein and neurotoxin residues, and the molecular conformation, which all can play significant roles in the neurotoxin effect, or, alternatively, its toxicity.

As a final outcome, there is inhibition of acetylcholine release, and the neurotoxin reduces muscle contraction, glandular secretion, and afferent signaling [50]. The result is chemodenervation.

BoNTs have a high affinity for Syt-II and SV2, and are expressed in motor neuron terminals. These isoforms are poorly expressed in the CNS hippocampus and in cerebral cortex terminals, where Syt-I, SV2A and SV2B are predominant [52,53]. The SV2C has only recently been discovered in small regions ranging from basal forebrain to brainstem [88]. The selective presence of different proteins and gangliosides in CNS and PNS can determine the sensibility to BoNTs subtypes within different targets. 

**Figure 4:**
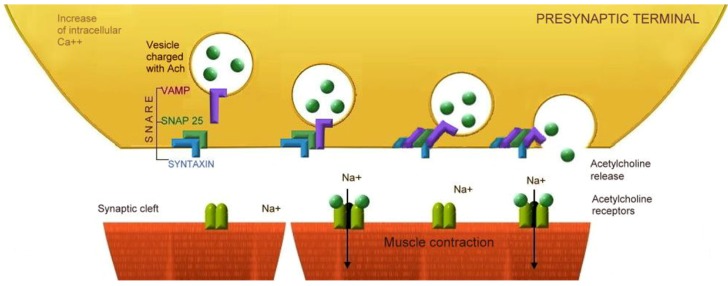
Neuromuscular Transmission: The role of the SNARE complex in acetylcholine release. The action potential travels along the presynaptic neuron and leads to intracellular calcium influx; this induces the adherence of vesicles charged with acetylcholine (Ach) to the presynaptic terminal membrane, where the SNARE protein complex participates in the vesicle exocytosis and release of Acetylcholine (Ach) to the neuromuscular junction. Ach molecules activate the postsynaptic nAChR (N1 Subtype), and open the central ion channel allowing a net inward flux of sodium whichleads to muscle contraction.

**Figure 5:**
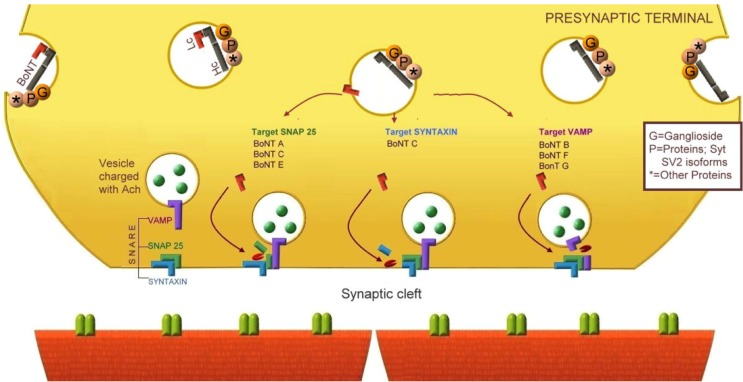
Botulinum neurotoxin blocking Ach release from the neuromuscular junction. The heavy chain (Hc) of neurotoxin binds to the presynaptic array of receptors exposed during vesicle recycling. The carboxyl terminal of Hc binds to specific gangliosides and proteins associated with the vesicle membrane (Syt and SV2 isoforms). Lateral movements of the exposed membrane increase the contact of BoNT with more proteins of the presynaptic receptor complex, and the molecule is endocytosed. The intravesicle acidification dissociates the Lc from Hc, and then the amino region of the heavy chain participates in the translocation of Lc into the cytoplasm to cleave specific proteins from the SNAREv and SNAREt complex according the neurotoxin subtype. BoNT/A, C, E target SNAP-25, BoNT/C also cleave syntaxin and BoNT/B, D, F, G target VAMP. The final result is blocking of the Ach release, preventing postsynaptic receptors activation.

Figure 4 illustrates the physiological neurotransmission, and Figure 5 illustrates the different mechanisms of botulinum neurotoxins A-G blocking the neurotransmission.

The inhibition of acetylcholine exocytosis by the BoNT is terminated by restoration of the SNARE protein complex. Although axonal sprouting and endplate elongation have been described, they appear to be transient phenomenon, and are not responsible for the cessation of the BoNT effect [89].

## 6. Current Commercially Available Neurotoxins

There are currently only two serotypes of Neurotoxin available—BoNT/A (OnabotulinumtoxinA, AbobotulinumtoxinA, and IncobotulinumtoxinA) and a BoNT/B, patented by RimabotulinumtoxinB (Table 4). Each brand has been approved by the US FDA to treat specific pathologies (Table 5). Every commercial brand of BoNT should be titrated by the clinician as an individual drug, and should be used for specific symptoms. The doses are not interchangeable between available serotypes, and between each formulation. Each formulation of BoNT/A, for example, is not interchangeable with another dose of BoNT/A if from a different formulation [90]. 

**Table 4:** Main commercial botulinum neurotoxins formulation profile [91].

Physiochemical Characteristics of BoNT formulations				
	OnabotulinumtoxinA (BOTOX^®^)	AbobotulinumtoxinA (DYSPORT^®^)	RimabotulinumtoxinB (MYOBLOC^®^)	IncobotulinumtoxinA (XEOMIN^®^)
Date introduced	1989	1991	2000	2005
Serotype	BoNT A	BoNT A	BoNT B	BoNT A
Total weight kDa	900	>500	700	150
Excipients	Sodium chloride	Lactose	Sodium chloride	Sucrose
	Albumin	Albumin	Sodium succinate	Albumin
			Albumin	
Final formulation	Vacuum dried	Freeze	Solution	Freeze dried
pH value	7	7	5.6	7

**Table 5:** Uses of neurotoxins: ON and OFF label indications [4,5,6,90,92].

Trading name^®^ and Non proprietary name	Year	FDA Approved	OFF Label Indications
BOTOX^®^ OnabotulinumtoxinA Units/vial: 50, 100, 200	1989	Strabismus Blepharospasm	
		Disease related to facial nerve	Focal hand dystonia
	2000	Cervical dystonia	
	2002	Glabellar lines	
	2004	Primary axillary hyperhidrosis	Lower Limb Spasticity
	2010	Chronic Migraine Upper limb spasticity	Myokymia
	2011	Hyperactive bladder	
DYSPORT^®^ AbobotulinumtoxinA Units/vial:300, 500	2009	Cervical dystonia Glabellar lines	Tensional Headache
			Head tremor
XEOMIN^®^ IncobotulinumtoxinA Units/vial:50, 100	2010	Cervical Dystonia	
		Blepharospasm	
	2011	Glabellar lines	Intractable Hyperkinesias
MYOBLOC^®^/NEUROBLOC^®^ RimabotulinumtoxinB Units/vial:2500, 5000, 10000	2010	Blepharospasm	
		Cervical Dystonia	
	2011	Glabellar lines	

After BoNT injection, clinical effects can be detected at approximately one week following injection, and the peak benefit is commonly observed approximately two weeks following injection. The timing of injection sessions varies by diagnosis, but is typically every three to four months, and booster injections before the next visit are not recommended because of the fear of neutralizing antibodies. The BoNTs are typically diluted in saline without preservatives. The amount of dilution, dosages and injection patterns can vary according the experience of the specialist, and the experience of the center. RimabotulinumtoxinB does not require dilution [93]. The reported side effects of the neurotoxins mimic botulism and may include blurred vision, eyelid drooping, dry mouth, dysphagia, muscle weakness, and rarely respiratory impairment [94,95,96]. Symptoms are usually focal and transitory, lasting no more than twelve weeks [93]. Other local side effects can include injection site hematomas, infections and pain. It should be noted that side effects directly correlate to the region injected. Autonomic reactions have been reported in more than one third of patients with cervical dystonia treated with BoNT/A [97], and autonomic resactions have been reported to be more frequent after BoNT/B injection compared with BoNT/A [98,99].

## 7. Immunogenicity

**Figure 6:**
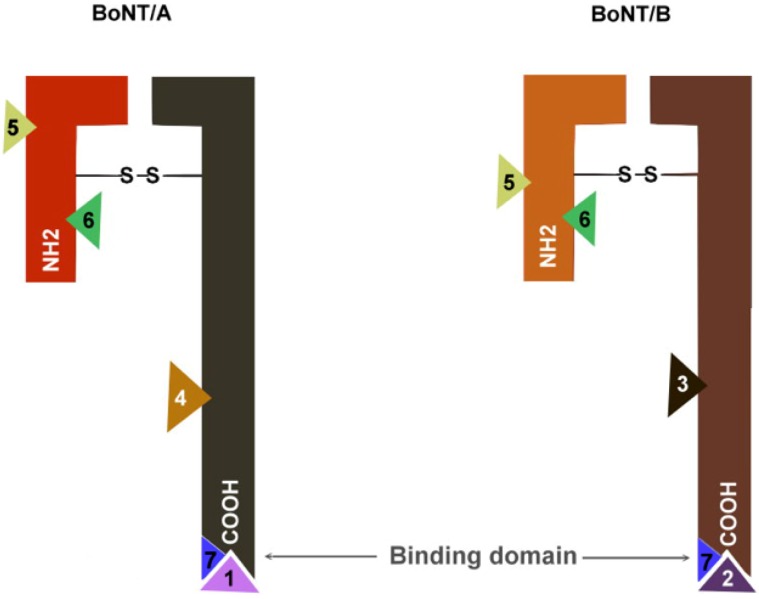
Representation of neutralizing and non-neutralizing antibodies against BoNT/A, and BoNT/B [106]. Neutralizing antibodies against BoNT/A <1> and B <2> affect neurotoxin heavy chain carboxyl terminal (Hcc), preventing its binding to presynaptic cells. Antibodies lacking neutralizing effects against BoNT/A <4> and BoNT/B <3> do not affect neurotoxin binding. According to somestudies, cross antibodies can recognize BoNT/A and BoNT/B <5,6>, but they do not affect neurotoxin binding [106]. A recent study reported neutralizing cross-reacting antibodies between neurotoxin serotypes <7> [107].

Patients who do not benefit from BoNT are considered primary non-responders. This occurs in about 30% of patients with cervical dystonia (CD) as a result of inadequate dosing, inaccurate muscle selection or inaccessible muscles. In contrast, secondary unresponsiveness to BoNT occurs in 16% of CD patients, of which 35% have antibodies against BoNT by the mouse neutralization assay [100]. Later, studies with AbobotulinumtoxinA and OnabotulinumtoxinA in CD patients reported ranges of neutralizing antibodies from 4% to 17% depending of the formulation used [97,101,102,103]. Factors which increase the risk of immunogenicity include large molecules of non-human origin and aggregated forms of the protein. The large size of the complex and the neurotoxin subunits increases the chance of antibody development against the BoNT preparation [104]. However, only a fraction of patients present with antibodies which can block neurotoxin effects (Neutralizing antibodies, Figure 6). Recent studies have reported the generation of neutralizing antibodies which act against epitopes from neurotoxin Hc and also from Lc [105].

The development of antibodies also depends on antigenic extrinsic factors; this includes the BoNT formulation, its physiochemical properties, the protein load, and the doses used per treatment cycle. The total cumulative doses, and the repeated treatment with booster doses (besides the regular three-month injections), could potentially increase the risk of antibody development [108]. Finally, host genetic factors may also be contributory [106].

When the OnabotulinumtoxinA formulation contained 25 ng of protein complex per 100 U of neurotoxin was administered to patients, 4%–17% were reported to develop a neutralizing antibody. The protein content has subsequently been decreased by the manufacturers to 5 ng per 100 U of neurotoxin (in 1998), with a reduction in the rate of neutralizing antibody production to 1.2% [109]. The incidence of neutralizing antibodies using AbobotulinumtoxinA has been reported as 0%–3%. The rate of neutralizing antibody with RimabotulinumtoxinB was reported as 10% at one year, and 18% at 18 months [110]. While reported in the literature, these results have not been replicated. 

The IncobotulinumtoxinA, which is free of proteins complexes, has not stimulated antibody development in rabbits when compared to OnabotulinumtoxinA and AbobotulinumtoxinA [111]. Additionally, there is a study in patients with upper limb spasticity that revealed no neutralizing antibodies following injections of IncobotulinumtoxinA or a placebo [112]. Approximately 1% of 1080 subjects from an IncobotulinumtoxinA development program presented with neutralizing antibodies; however, each of the patients had been previously exposed to BoNT which contained complexing proteins [22]. 

More comparative studies and longer term follow-up will be necessary to clarify the advantages and disadvantages of BoNTs lacking protein complexes compared with the conventional BoNTs preparations. Each neurotoxin can potentially generate an antibody response and produce cross-reacting antibodies, based on similar antigenic structures. Cross-reacting antibodies can recognize BoNT/A, and BoNT/B, but may not affect neurotoxin binding [106]. However, a recent study identified two clonally related human monoclonal antibodies designated as 1B18 and 4E17 with potential neutralizing effects. Antibody 1B18 was reported to bind BoNT/A, and BoNT/B, and antibody 4E17 to bind BoNT/A, B, E and F. Both antibodies bound to a conserved epitope at the tip of the BoNT translocation domain. The immunoglobulin G constructed from variants of 1B18 was found to neutralize BoNT/B *in vivo*, and 4E17 were found to neutralize *in vivo* BoNT/E, suggesting the functional importance of this epitope in the intoxication pathway [107].

An antibody neutralization experiment was performed in mice and the equivalent amount of BoNT/A1 or BoNT/A5 antibody was directed against mice injected with BoNT/A1. The mice injected with BoNT/A5 antibodies died faster than those injected BoNT/A1 antibodies [35], suggesting different antibody neutralization profiles for BoNT/A1 and BoNT/A5. A major improvement in the understanding in BoNT immunogenicity could contribute both to an optimization of BoNT treatment, and to development of better human vaccines for botulism.

## 8. Future Challenges and Opportunities for Use of Botulinum Neurotoxins

The specific function of acetylcholine at a particular cholinergic synapse is largely determined by its Ach receptor subtype [47], classified as nicotinic and muscarinic. These receptors project extensively throughout the central nervous system, innervating a broad range of structures within the brain; however, their mutual interactions remain unknown. The Nm subtype of nicotinic receptor dominates at the neuromuscular junction, and the Nn subtype is known to be present at autonomic ganglia, and in the central nervous system. At the same time the muscarinic receptors (M1 to M5) are distributed in a variety of locations; the muscarinic receptor M1 is found in pyramidal cells from brain cortex and hippocampus, and this receptor is mainly excitatory [113]; M2 is expressed in the nucleus basalis, occipital cortex and in lower levels in hippocampus, caudate and putamen [114], and also seems to participate in glutamatergic and gabaergic transmission; M3 has a similar distribution to M1, but with lower expression; M4 receptors are highly expressed in caudate and putamen and are associated with dopaminergic receptors [115], and the M5 receptor is expressed in low levels in the hippocampus, substantia nigra and ventral tegmental area [116]. Ach neurotransmission in the CNS has been implicated in the pathophysiology of several psychiatric and neurological diseases including schizophrenia, bipolar disorder, substance abuse, Alzheimer’s disease and Parkinson’s disease [117]. Therefore, an improved understanding of the Ach network and its modulator role in the nervous system could lead to the engineering of new neurotoxins to address pathologies beyond the current indications.

Biomolecular research is continually expanding the study of neurotoxins’ serotypes, subtypes and variants. It was discovered recently that there was a specific strain of *C. botulinum* which produced a DC mosaic botulinum neurotoxin formed by 2/3 of BoNT/D and 1/3 of BoNT/C [118]. This new neurotoxin was called BoNT/DC, and resulted in high lethality in mice (1.1 × 10^−9^ LD50/mg protein) compared with other types of neurotoxins [70]. This lethality could be related to particular ganglioside binding properties of BoNT/D and BoNT/C. The ganglioside binding pattern is also influenced by the conservative motif SXWY. Most of the botulinum neurotoxins which do not have the SXWY motif, bind to gangliosides using only the ganglioside binding site (GBS). BoNT/D and C do not have a SXWY motif; However, BoNT/D can recognize gangliosides using either GBS or a protein binding site (PBS), and BoNT C can recognize gangliosides through GBS and also has an auxiliary contribution of a ganglioside binding loop (GBL). The ganglioside recognition seems to lead to the binding activity of the neurotoxin to the specific cell [71].

The progress in biomolecular technology has made designs of recombinant neurotoxins possible. For example, replacing the amino acid histidine in the position 1241 of BoNT/F with a corresponding lysine residue of BoNT/E increases the affinity of the modified BoNT/F for GD1a. This change also allows a further ability of this recombinant neurotoxin to bind ganglioside GM1a, something BoNT/E could not do [71]. Another significant target for molecular engineering is the recognition of SNARE protein substrate by neurotoxins. There can be a potential manipulation of the neurotoxins main pockets and Lc residues in order to increase the affinity to the specific SNARE protein target. The enhanced understanding of the molecular structure and the conformation of the different neurotoxin serotypes could help researchers to design new hybrid toxins. These capabilities will further improve, as the science continues to expand.

Centrally administered BoNTs can block the release of other neurotransmitters including glutamate, glycine, noradrenalin, dopamine, serotonin and neuropeptides [17]. BoNT/A and BoNT/E have been suggested as possible candidates for epilepsy treatment because of their excitatory/inhibitory signal modulation effect, with predominant sensibility from the glutamatergic terminals as compared with gabaergic terminals [48]. BoNT/E has been tested to control epileptic discharges in a mouse model of mesial temporal lobe epilepsy. In this model, it was observed that BoNT administration during chronic seizure phases reduced the seizure frequency (ANOVA and Holm-Sidak test *p* < 0.05); however, its benefit only lasted for 21 days [119]. Other studies have suggested a neuroprotective role for BoNT/E, before or shortly after status epilepticus [120,121,122]. Neurotoxins are also interesting candidates for pain management, e.g., BoNT/A has been proposed as an analgesic because of a proposed reduction in the inflammatory phase of pain. BoNT/A may inhibit the release of certain nociceptive mediators such as substance P [123], calcitonin gene-related peptide (CGRP) [124], and glutamate [125]. The proposed mechanism of neurotoxin action on nociception has been described as blocking neurotransmitter release at the peripheral nerves, preventing pain perception and also by preventing peripheral sensitization [50]. BoNT may also prevent an indirect central sensitization mechanism. BoNT has subsequently been considered as a candidate for chronic inflammatory pain treatment, and BoNT/A is suggested as the most suitable serotype based on its stable intracellular proteolytic activity [126]. BoNT has been approved by the FDA for chronic migraine treatment [2], and has been used off-label in tension headaches by some practitioners. BoNT is also being studied in trigeminal neuralgia. A recent publication suggested that BoNT/A preferentially targeted C fibers, blocked neurotransmitter release, and subsequently reduced pain, neurogenic inflammation, and the cutaneous heat pain threshold. These findings were documented in a capsaicin induced trigeminal neuralgia human model [127]. Some small studies treating trigeminal neuralgia patients with BoNT also revealed clinical benefits [128]. A randomized, double-blind placebo controlled study with BoNT/A was performed in a mouse trigeminal neuralgia pain model [129]. The results suggested that BoNT/A was effective in preventing inflammatory pain up to eight days after the first treatment; however, the effect was not reproduced during the second dose. Randomized and controlled studies in humans with trigeminal neuralgia are needed to assess the neurotoxins’ potency and duration in pain treatment. Furthermore, a recent study found that when selectively targeting syntaxin, the neurotoxin can block synaptic release in neurons, and norepinephrine release in neuroendocrine cells [130]. 

The development of secondary resistance has resulted in increasing attention being given to the immunogenicity of the associated proteins, the neurotoxin size and configuration, and the influence of serotype. Designing new neurotoxin molecules has the potential to expand the possibilities for treatment of resistant patients, expansion of clinical indications, and limitation of side effects. An initial target may be the presynaptic specific gangliosides and proteins, and the exposed vesicular membrane. A second target may be the specific affinity for different BoNTs serotypes to the SNAREt and SNAREv complex.

Modifying the sequence of carboxyl terminals on the neurotoxin Hc to match the predominant gangliosides or SNAREv proteins, and future studies of the intervention in the Lc translocation and proteolytic stage could contribute to the potency of neurotoxin effect in refractory patients. Additionally, identifying SNAREt isoforms and their localizations in CNS and PNS could aid in increasing the specificity of neurotoxin uses and could provide better control of adverse effects. However, this has proven to be a complex field, with evidence that there are multiple cofactors important in the presynaptic binding, as well as in the SNARE cleavage. Another important aspect which needs further study is the mechanism of neurotoxin penetration in different tissues, for example, hyperactive neurons of epileptic foci could over-expose receptors associated to the high rate of exocytosis and endocytosis.

## 9. Conclusions

An ideal botulinum neurotoxin preparation should be stable in the environment, have low immunogenicity, have a specific function in determined cells, and have an adequate potency with controllable effects, long-lasting benefits, and few side effects. The ability to design neurotoxin recombinant mixtures may also enhance Hc terminal binding to the presynaptic protein array, aiding in the control of specific vesicular and *trans*-membrane SNARE proteins. Recombinant neurotoxin molecules are currently being studied in basic sciences laboratories, and these may play a role in botulinum neurotoxin resistant cases associated with antibody production. The potential clinical uses for botulinum neurotoxins are still largely undefined, but there is expanding interest in the potential role of neurotoxin in multiple disorders. There will likely be many possibilities for BoNT use beyond movement disorders, pain and other current indications. The understanding of the pathophysiologic mechanisms by which neurotoxins affect specific neuronal populations, and the differential effects seen with different serotypes, will aid us in tailoring treatment for each diagnosis and each patient’s individual needs.
